# Self-organization of common good usage and an application to Internet services

**DOI:** 10.1093/pnasnexus/pgaf374

**Published:** 2025-12-01

**Authors:** Diogo L Pires, Vincenzo Mancuso, Paolo Castagno, Marco Ajmone Marsan

**Affiliations:** Copenhagen Center for Social Data Science, University of Copenhagen, 1353 Copenhagen K, Denmark; Department of Engineering, University of Palermo, 90133 Palermo, Italy; IMDEA Networks Institute, 28918 Leganes (Madrid), Spain; Computer Science Department, University of Turin, 10125 Turin, Italy; IMDEA Networks Institute, 28918 Leganes (Madrid), Spain; Department of Electronics and Telecommunications, Polytechnic University of Turin, 10129 Turin, Italy

**Keywords:** common goods, complex systems, hybrid systems, mobile networks, multiagent reinforcement learning

## Abstract

Natural and human-made common goods present key challenges due to their susceptibility to degradation, overuse, and congestion. We explore the self-organization of their usage when individuals have access to several available commons but limited information on them. We propose an extension of the Win-Stay, Lose-Shift (WSLS) strategy for such systems, in which individuals use a resource repeatedly until they are unsuccessful and then shift randomly. This simple and completely decentralized strategy promotes balanced resource use based solely on individual experience, as it does not require communication between individuals nor a governing institution to coordinate usage. Selective individuals who retain information on their usage and accordingly adapt their tolerance to failure in each common good improve the experienced quality for all individuals in the population. Even in hybrid populations of adaptive and nonadaptive individuals, the WSLS strategy allows self-organization into an ideal distribution with equal experienced quality across common goods. We apply this strategy to the server selection problem faced by mobile users when accessing Internet services. Realistic simulations demonstrate its success, scalability, and robustness to dynamic system conditions. Furthermore, the WSLS strategy can be used to understand animal dispersal on grazing and foraging land, to propose solutions to operators of systems of public transport and other technological commons, and to address problems of common good usage in social systems through decentralized governance rather than control-oriented policies.

Significance Statement Common goods are shared resources, both natural and human-made, such as groundwater, land, transport, or technological infrastructure, whose usage reduces their availability or quality to others. We propose a simple usage strategy for individuals with different commons available to them, inspired by observed behavior: use the same resource while satisfied, shift to a different one when dissatisfied. This remarkably simple and fully distributed strategy leads to a successful distribution of the users over the available resources, avoiding disproportionate usage. Our findings provide theoretical insights into the distribution of usage of land and other natural resources as well as applicable solutions for managing socio-technical commons that address the question resorting to decentralized governance rather than control-oriented policies.

## Introduction

Common goods are resources that are accessible to multiple individuals where one individual’s use reduces the amount available to others (1). These typically include natural resources such as groundwater basins, grazing land, forests, air quality, and fisheries. However, their challenges are sometimes parallel to those of human-built resources available for collective use, such as roads, public transport systems, and Internet services. Shared usage of such resources is pervasive in social systems making their study central to economics, social and life sciences. Given the finite nature of commons, several challenges arise from their usage, which under uncoordinated action may lead to the “tragedy of the commons” as described by Hardin in Ref. (2). As a result, the governance of these shared resources has become a crucial issue, extensively studied by Elinor Ostrom, in for example Ref. (1), whose work in this area earned her the Nobel Memorial Prize in Economic Sciences.

Individuals often have several commons available to them that may fulfill the same need. This raises questions on how such systems can attain a sustainable distributed consumption and avoid scenarios of disproportionate usage, over-consumption and depletion of one of the commons while others remain available. In the context of grazing, foraging, and hunting, both animals and humans must decide whether to remain in a partially exploited land or move in search of new resources. These dynamics have contributed to the evolution of nomadic patterns, both in hunter–gatherer and pastoralist societies. Moreover, parallel problems emerge in industrialized societies. For instance, individuals have to choose daily which form of public transport to take or which road to drive on; institutions managing water distribution may need to choose which water resources to use; fishing companies have to decide the areas at which they will fish; and devices connected to mobile networks, such as mobile phones, have to choose to which computing facilities they will send their requests. The quality or availability of each of these resources decreases with increasing number of individuals simultaneously using them, therefore conferring them similar properties.

The ideal free distribution (IFD) theory was originally developed in Ref. (3) in the context of animal territorial behavior. It explores individuals distributing themselves across different resource patches to maximize their own benefits, assuming perfect knowledge and no movement costs. Under the IFD, individuals spread in a way that equalizes the experienced quality across all used resources. The fact that the IFD strategy constitutes an evolutionarily stable strategy was proven in Ref. (4). However, when individuals have reduced information on the current experienced quality in all the available commons, achieving distributed usage across them may be challenging. Individuals may perform back and forth movements between resources to directly assess their quality, as explored in Ref. (4). However, the system becomes more complex when the quality of a resource is not instantaneously measurable. In the aforementioned systems, coordination would require constant communication between individuals, or a governing institution to direct individuals on which commons to use. These options are often unfeasible or, at the very least, costly.

In this work, we explore a simple and fully decentralized strategy that enables self-organization towards the ideal distribution of common good usage. The Win-Stay, Lose-Shift (WSLS) strategy was originally introduced in the context of the iterated prisoner’s dilemma (5) and proved highly effective in Axelrod’s competitive tournaments (6), outperforming established strategies such as Tit-for-Tat (7, 8). In WSLS, individuals repeat an action following a successful outcome, otherwise shift to an alternative action. Its underlying principle can be traced back to Robbins’ ideas (9), which laid the foundations for multiarmed bandit methods. The WSLS strategy rapidly corrects occasional errors, exploits unconditional strategies, and requires only knowledge of the previous outcome. Owing to these properties, WSLS has been extended to the study of conditional movement in this dilemma (10), and later to public goods dilemmas (11). The coevolution of WSLS movement rules with cooperation has been proved in Refs. (12, 13), and applied to the study of social agglomeration and cohesion (14, 15), social mobility (16), and spatial pollution (17). However, public goods dilemmas involve individuals incurring personal costs to produce a shared benefit, differing in nature from common goods dilemmas, which concern the consumption of finite, preexisting resources subject to depletion (1, 18).

We propose an extension of the WSLS strategy to systems of usage and consumption of common goods: individuals consume a particular common good until they are unsuccessful or their experienced quality falls below a threshold, at which point they shift to a different good at random. We show that the resulting dynamics lead to a more balanced distribution of common good usage without the need for individuals or central institutions to store, transmit, or process any information. This decentralized, noncontrol-based mechanism enables populations to coordinate usage patterns efficiently. When individuals have selective tolerance to failure across different common goods, the system self-organizes onto an ideal usage distribution, where experienced quality is equalized across all actively used goods. Remarkably, individual efforts to reduce experienced failure rates lead to improved outcomes for the entire population. This striking result also emerges in hybrid populations consisting of both selective and nonselective individuals. Throughout the article, we show practical applications of these results, particularly focusing on individuals accessing Internet services through mobile networks. We first formalize this as a common good usage system, showing that WSLS quickly and efficiently distributes usage over the available servers in a mobile network. We then propose a method for adaptive tolerance to failure based on individually collected information on the different common goods, and demonstrate its effectiveness in realistic conditions, including quickly changing environments. The usage of the WSLS strategy can be extended to understand other systems, such as population distribution over grazing or foraging land, or the management of complex socio-technical infrastructures like public transport networks and shared digital services. In all cases, a simple decentralized strategy grounded in individual experience can replace control-oriented governance mechanisms, offering scalable and adaptive alternatives for managing common goods.

## Win-Stay, Lose-Shift good

**Fig. 1.**
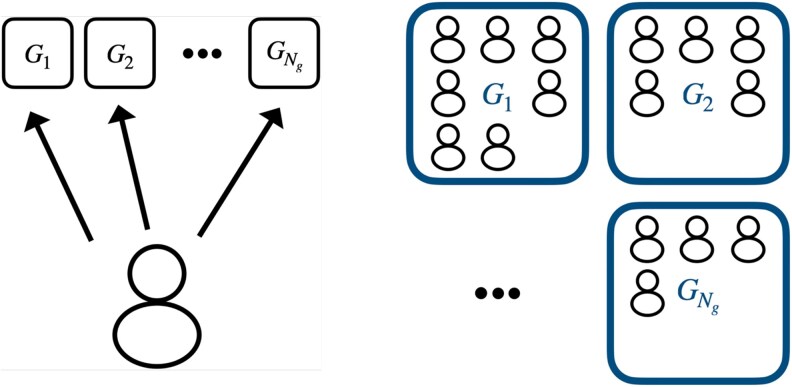
Representation of a system of common good usage. On the left, individuals can choose which of the $N_{g}$ goods they will use without any information besides their individual experience. On the right, the distribution of the population of individuals over the available goods.

We consider a population of $N_{u}$ users with an available set of $N_{g}$ common goods which are denoted $G_{i}$, with $i=1,2,\ldots ,N_{g}$. This system is represented in Fig. 1. We denote as $Q_{i}$ the quality of common good $G_{i}$. The quality may relate to a quantifiable probability of having a failed or unsatisfactory attempt to use the good $Q_{i}=1-P_{i}^{\left(F\right)}$, where $P_{i}^{\left(F\right)}$ is the failure probability of good $G_{i}$, holding a value between 0 and 1. We consider the cases where probability of failure increases, and therefore quality decreases, with the number of current simultaneous users $n_{i}$ of $G_{i}$. Note that $\sum_{i=1}^{N_{g}}n_{i}=N_{u}$. A failed attempt might happen due to reduced availability, overcrowding, general lower quality of experience, or active competition with other users. As mentioned in the introduction, some examples of these goods can be land for grazing or foraging, fishing, or hunting areas, water supply systems, means of transportation, technological goods, or Internet services such as those offered by mobile network operators.

Let us consider that the population is fully distributed and individuals have minimal information. They get no information about the characteristics of the common goods from neither one another nor central institutions, e.g. operators. They are only informed by the direct perception of the quality of the used good, also lacking information about the current number of users of the good.

In this context, we introduce an extension of Win-Stay, Lose-Shift strategy to common good usage. Individuals do not interact directly with each other, but only with the good they have chosen to use. Under WSLS strategy, individuals initially choose one of the available goods at random and stay there until they have a failed or unsatisfactory attempt of use. When the failed event occurs, they shift to one of the other goods at random. If in the particular system considered, individuals cannot fail to use the commons, and instead they just have a lower experienced quality, then consider that they may set their own probability of shifting proportional to the negative of the experienced quality of the good.

Consider a large enough population of individuals using the described WSLS strategy. Each individual attempts to use the good of their choice at an average frequency of $\lambda_{u}$ attempts per unit time. Let us assume that the quality of the good they are using changes slowly, and that their usage may have only an infinitesimal relative effect on the current number of users $n_{i}$ of each good, given the large size of the population. As it is typically done in population dynamics, this system can be modeled through a set of differential equations describing the rate of change in the distribution of individuals of the population, in this case, using each common good:


(1) $$\dot{n_{i}}=-\lambda_{u}\cdot n_{i}\cdot P_{i}^{\left(F\right)}(n_{i})+\frac{1}{N_{g}-1}\sum_{\;j\neq i}\lambda_{u}\cdot n_{\;j}\cdot P_{j}^{\left(F\right)}(n_{j}),$$

where $n_{i}$ is approximated to a continuous variable.

The first term on the right hand-side corresponds to the rate at which individuals have failed usage attempts and leave the common good $G_{i}$. The second term corresponds to the rate at which individuals have failed attempts at using other common goods and shift to $G_{i}$. This leads to the following equilibrium equations:


(2) $$n_{1}\cdot P_{1}^{\left(F\right)}(n_{1})=n_{2}\cdot P_{2}^{\left(F\right)}(n_{2})=\ldots =n_{N_{g}}\cdot P_{N_{g}}^{\left(F\right)}(n_{N_{g}}).$$

In the next section, we will illustrate an application of the WSLS strategy through a realistic system of common good usage: individuals accessing Internet services through mobile networks. We will show the alignment with the equilibrium derived above. Despite concluding it presents a better distribution of usage over the available resources than if individuals used them at equal rates, the outcome remains less-than-ideal as resources with lower usage capacity are overused and those with higher capacity remain underused. We later introduce a definition of the ideal usage distribution, alongside an adaptive WSLS strategy that achieves it.

## Application to Internet services

**Fig. 2.**
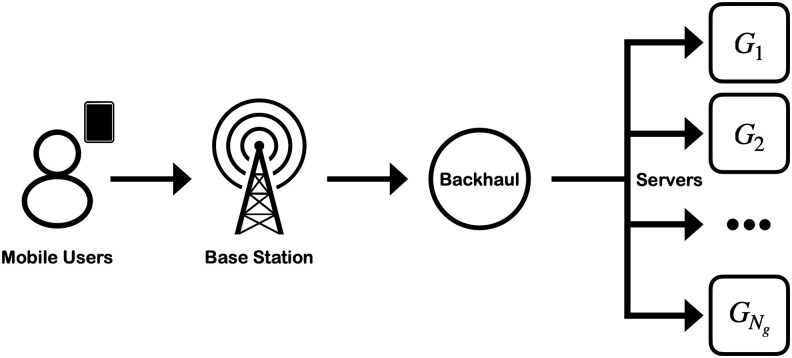
Server selection for Internet access as a system of common good usage. A population is constituted of active mobile users who connect to the network through a base station. They have their connection attributed through the backhaul to their chosen server $G_{i}$ out of $N_{g}$ available options.

Mobile networks are wireless communication systems that enable users to connect and exchange data, such as voice, text, and Internet services through interconnected base stations. Connecting to such systems allows users to perform computations on in-network computing facilities, i.e. servers, which in turn are essential for supporting the above mobile services. In these cases, active mobile users submit frequent requests to the network, which are processed in the base stations and the associated network backhaul, and are then routed to a server for computing. The servers have different characteristics and their performance decreases as the number of concurrent users increases. The users can often select the server which will process their requests, although with very limited information on them. This leads to the *server selection problem*, where the extension of the WSLS strategy to common good usage could provide valuable insights. Figure 2 shows a schematic representation of this system.

The server selection problem (also called *computation offloading* or *request routing*) has been gaining increasing relevance with the deployment of the new generations of mobile networks, which exploit servers in different network locations for both the management of network resources and the satisfaction of user service requests. The server selection problem was previously studied using approaches different from the one we consider in this work (see for example Ref. (19) for a recent survey and Ref. (20) for an early seminal result). The new approach we introduce is both fully distributed and more generally applicable than the ones in the existing literature.

Servers exhibit diverse characteristics, including service latency and computing power. Latency is defined as the delay between sending a service request and getting the corresponding response. Its main components are the time $d_{i}$ for a request to travel from the user to the server and the processing delay at the server. Computing power refers to the ability of a server to process tasks quickly and handle large amounts of data, which can be quantified by the number of requests per unit time they have the capacity to serve, denoted as $\mu_{i}$. We further denote the total system capacity as $\mu =\sum_{i}\mu_{i}$, representing the total number of requests it can serve per unit time. The system workload is denoted as $\rho =N_{u}\cdot \lambda_{u}/\mu$ and represents the ratio between the population request rate and the system capacity.

These two server characteristics determine the server-specific failure probability of submitted service requests and its dependence on the number of concurrent users. Each server immediately processes requests that arrive to find it idle, and queues requests that arrive when the server is busy. Due to a finite buffer size, some requests can be lost because they arrive when the server’s buffer is full—this is called a loss event and has probability $P_{i}^{\left(L\right)}(n_{i})$ at server *i* when $n_{i}$ users are accessing it. Others are discarded by the users when the results of the computation are returned to the requesting user too late to be useful—this is called an excessive delay event and has probability $P_{i}^{\left(D\right)}(n_{i})$. Both cases lead to failed attempts at using the server. Therefore, the failure probability can be calculated as $P_{i}^{\left(F\right)}(n_{i})=P_{i}^{\left(L\right)}(n_{i})+(1-P_{i}^{\left(L\right)}(n_{i}))\cdot P_{i}^{\left(D\right)}(n_{i}).$ Based on the characteristics of each server, both the loss probability and the time delay distribution can be calculated analytically using standard queuing theory results as in Ref. (21), which are briefly described in the “Materials and methods”. We assume the outcomes of any two submitted requests to be independent and have failure probability that change slowly. This allows us to describe a population using the WSLS strategy in such a system through Eqs. 1 with equilibrium condition 2.

**Fig. 3.**
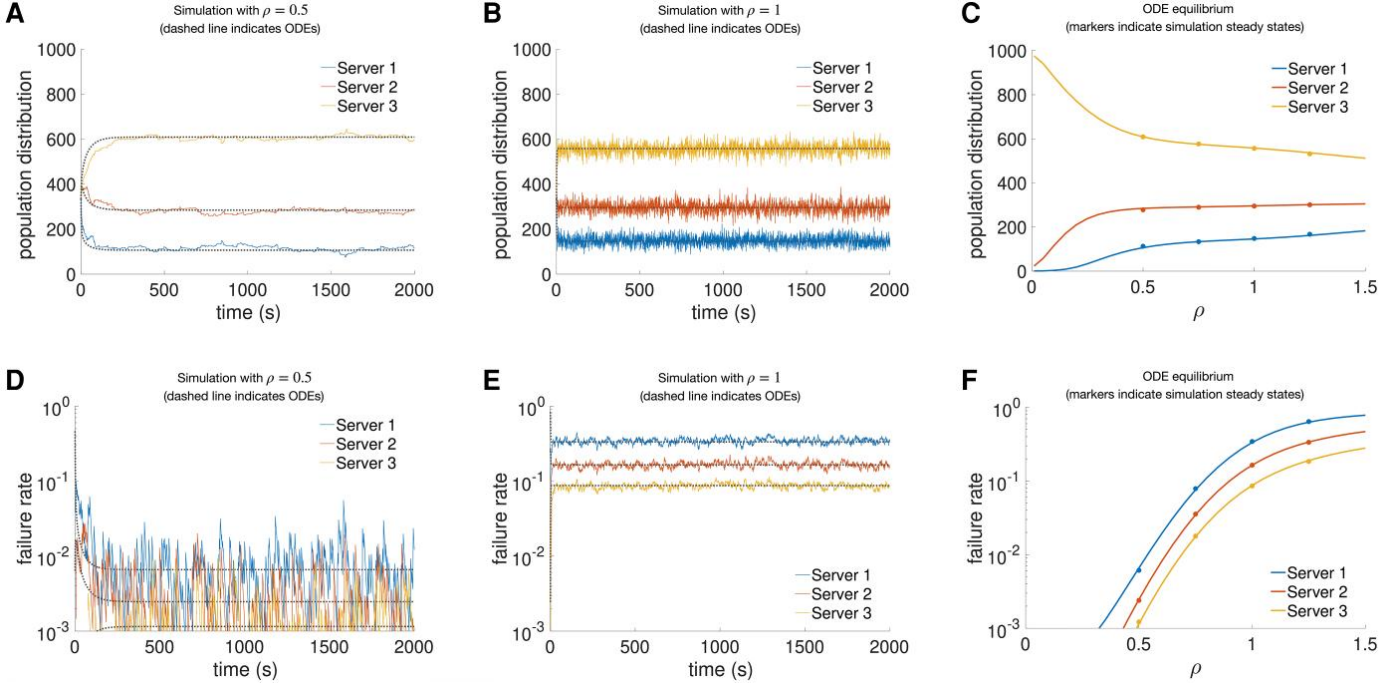
Simulation of a population of 1,000 users using a WSLS strategy accessing three servers with different capacity and delay values. A, B) show the evolution of the population distribution, while D, E) depict the server-specific failure probabilities, for two system workload values. C, F) compare the values obtained from the ODE equilibria with those from the simulation steady states over a wider range of system workload values. See “Materials and methods” for details on the simulator and the used parameters.

To validate the theory development, we compare the evolution of the ODEs with the results obtained with realistic simulator parallel to the one used in Refs. (21, 22), which is further explained in the “Materials and methods”. The resulting evolution of the population distribution and server-specific failure probability are presented together with the differential equation results in Fig. 3. Furthermore, the values from the ODE equilibria are compared to those of the simulation steady states. These are obtained for a system with three servers sorted from lowest to highest capacity and delays, and for a wide range of system workload values (*ρ*). The used parameters are defined in the “Materials and methods”. The results obtained through the simulator align with what was predicted by the analysis of the dynamical system originally proposed.

In particular, we observe that under all considered system workload values, the result of the evaluation shows the population distribution evolving to the theoretical value given by Eq. 2. In this equilibrium, a server with higher capacity holds more users and exhibits lower server-specific failure probability than other servers. This suggests that the equilibrium slightly overflows servers with lower capacity, but because their usage rate is overall lower, this has a low impact on the overall system probability of failure. In line with what is mentioned in the previous section, the obtained equilibrium represents an improvement when compared to equal usage rates across commons, but it is still less-than-ideal from the point of view of resource management, as some servers are disproportionately used leading to higher failure rates.

Furthermore, convergence to the equilibrium and alignment with the ODE result is almost immediate under higher workload. There seems to be a slight delay between simulation and ODE results under lower workload, possibly due to the lower overall failure probability and the longer time required to saturate the servers. Stochastic oscillations around the population distribution equilibrium are more pronounced under higher workloads, whereas oscillations in the server-specific failure probabilities are greater under lower workloads.

## Introducing selective tolerance to common good failure

We further consider a heterogeneous population with $N_{t}$ types of individuals with subpopulations of size $N_{u}^{\left(1\right)},N_{u}^{\left(2\right)},\ldots ,N_{u}^{\left(N_{t}\right)}$, with $\sum_{k}N_{u}^{\left(k\right)}=N_{u}$. Each type *k* of individual has a set of tolerance (or threshold) values $T_{i}^{\left(k\right)}$, which dictate the amount of failures they will accept with common good $G_{i}$ before shifting to a different one. We make the simplifying assumptions that the outcomes of any two usage attempts are independent and have failure probability $P_{i}^{\left(F\right)}(n_{i})$, and that this value changes slowly with time. In this case, the number of attempts an individual makes until the number of failures achieves their tolerance value should follow a negative binomial distribution with average value $T_{i}^{\left(k\right)}/P_{i}^{\left(F\right)}(n_{i})$. Therefore, the probability that a randomly chosen attempt of usage by an individual of type *k* leads to shifting is equal to $P_{i}^{\left(F\right)}(n_{i})/T_{i}^{\left(k\right)}$. Considering large subpopulations of types, we again describe the approximately continuous changes in the subpopulation distribution $n_{ik}$, i.e. the number of users of type *k* using each common good *i*, in differential terms:


(3) $$\dot{n_{ik}}=-\lambda_{u}\cdot n_{ik}\cdot \frac{P_{i}^{\left(F\right)}(n_{i})}{T_{i}^{\left(k\right)}}\;+\;\frac{1}{N_{g}-1}\sum_{\;j\neq i}\lambda_{u}\cdot n_{\;jk}\cdot \frac{P_{j}^{\left(F\right)}(n_{j})}{T_{j}^{\left(k\right)}}.$$

The population will be at equilibrium when the following conditions are met for all types *k*:


(4) $$\frac{n_{1k}\cdot P_{1}^{\left(F\right)}\left(n_{1}\right)}{T_{1}^{\left(k\right)}}\;=\;\frac{n_{2k}\cdot P_{2}^{\left(F\right)}\left(n_{2}\right)}{T_{2}^{\left(k\right)}}\;=\ldots =\;\frac{n_{N_{g}k}\cdot P_{N_{g}}^{\left(F\right)}\left(n_{N_{g}}\right)}{T_{N_{g}}^{\left(k\right)}}.$$

Under a state of equilibrium, the different types of individuals will be distributed between the set of available commons depending not only on the probability of failure functions and the population size, but also on the values of tolerance to failure of the individuals in the population. The original equilibrium from Eq. 2 is recovered when each individual holds the same tolerance value under all common goods. We show in the Supporting Information and Figs. S1 and S2 that compared to the previous population, this only extends the timescale at which the system evolves.

The equilibrium given by Eq. 4 still represents a less-than-ideal scenario if the usage distribution leads to higher failure rates in some goods due to overuse or overconsumption, similarly to what can happen in the original dynamical equilibrium given by Eq. 2 and observed in the previous sections. We introduce Definition 1 of the ideal distribution with equalized quality, inspired by the ideal free distribution (3, 4), as an ideal organized distribution of common good usage.

Definition 1 For a given population size $N_{u}$, we denote $n^{*}=[n_{i}^{*}]$ respecting $\sum_{i}n_{i}^{*}=N_{u}$ as the ideal distribution with equalized quality between used common goods. We use index *a* for the subset of common goods which are used by the population under such distribution, i.e. those $G_{a}$ such that $n_{a}^{*}>0$. We use index *b* for the complementary subset of unused common goods, i.e. those $G_{b}$ such that $n_{b}^{*}=0$.

In this distribution, the subset of used common goods holds the same failure probability, therefore being equal to the experienced population average failure probability, denoted $P^{\left(F\right)}(n^{*})$:

(5) $$P_{a}^{\left(F\right)}\left(n_{a}^{*}\right)=P^{\left(F\right)}\left(n^{*}\right).$$

In the ideal distribution with equalized quality, the complementary subset of unused common goods will respect the following condition:

(6) $$\lim_{n_{b}^{*}\to 0}P_{b}^{\left(F\right)}\left(n_{b}^{*}\right)>P^{\left(F\right)}\left(n^{*}\right).$$

A self-interested individual looking to maximize the success of its usage of commons would avoid those with higher failure probabilities. In strategic terms, under a WSLS strategy with selective tolerance, they would increase their tolerance to failure for commons with lower failure probabilities and decrease their tolerance for higher probability ones. Due to the competing nature of the use of commons, lower usage of one of them decreases the failure probability at it. Therefore, self-interested individuals would have a positive impact on the overall system and push in the direction of equalized quality and failure probabilities between different commons, even if the impact of a single individual is negligible. This will be further elaborated in later sections by considering adaptive tolerance to failure. For now, let us start by noting that a population with one or more types of individuals *can* achieve the ideal distribution with equalized quality between common goods if individuals tune in their tolerance values accordingly. Theorem 1, proven in the “Materials and methods”, describes this result.

Theorem 1 The population distribution $n_{i}^{*}$ corresponding to equalized quality between used common goods is attainable by any population using a WSLS strategy if and only if they hold a set of tolerance vectors $T_{i}^{\left(k\right)}$ that respects

(7) $$\sum_{k=1}^{N_{t}}N_{u}^{\left(k\right)}\cdot \left(\frac{T_{i}^{\left(k\right)}}{\sum_{j}T_{j}^{\left(k\right)}}\right)=n_{i}^{*}.$$

A population using a WSLS strategy can always achieve the ideal distribution with equalized quality between common goods if they accordingly choose their selective tolerance to failure. Even though central coordination between individuals could lead to equalized quality, fully distributed populations composed of self-interested individuals might achieve the same by trying to minimize the failure probabilities of individual requests. We will explore this hypothesis by resorting to adaptive tolerance to failure later in this article.

Note that for any set of common goods, there might exist population sizes $N_{u}$ for which the equal performance between used common goods will exclude completely a subset of the commons. In this case, for the ideal distribution with equalized quality to be achieved, all types of individuals will necessarily have no tolerance to failure in that good $T_{i}^{\left(k\right)}=0$, meaning that they will move from it without submitting requests. However, if there are no such common goods, hybrid populations with both selective and nonselective individuals might be enough to achieve equalized quality.

## Hybrid systems of selective common good usage

Let us consider a system with only two types of individuals k=1,2. Individuals of type k=1 do not distinguish between common goods, thus being nonselective individuals with constant $T_{i}^{\left(1\right)}=T,\forall i$. Individuals of type k=2 have selective tolerance values towards common goods $T_{i}^{\left(2\right)}$. We denote the fraction of selective individuals as $\gamma =N_{u}^{\left(2\right)}/N_{u}$.

Applying Theorem 1 to the population defined by these parameters, we conclude that the ideal distribution with equalized quality is attained if selective individuals choose their tolerance to failure as to respect the following equations:


(8) $$(1-\gamma )\;N_{u}\cdot \frac{1}{N_{g}}+\gamma N_{u}\cdot \frac{T_{i}^{\left(2\right)}}{\sum_{j}T_{j}^{\left(2\right)}}=n_{i}^{*}.$$

Under conditions of equalized quality, certain common goods exhibit lower usage ($n_{i}^{*}$) compared to others. However, the original nonselective equilibrium, as expressed in Eq. 2 and recovered under γ=0, results in a less-than-ideal intermediate state: even though those commons have lower usage rates than others, the difference is insufficient to reach the ideal distribution, leading to higher failure probabilities on those commons. Consequently, selective individuals respecting Eq. 8 will correct this by avoiding commons which ideally would have lower usage and flock to the remaining ones.

However, the condition of equilibrium with equalized quality of Eq. 8 may only be fulfilled if *γ* is large enough. We denote the lowest usage of any of the common goods at equalized quality as $n_{\min}^{*}=\min_{i}(n_{i}^{*})$, which may be zero. The critical value $\gamma^{c}$ above which equalized quality can be attained is the one where selective individuals do not spend any time on the common good(s) corresponding to that minimum, i.e. $T_{\arg\;\min(n_{i}^{*})}^{\left(2\right)}=0$. Applying this to Eq. 8 replacing *i* by $\arg\;\min_{i}(n_{i}^{*})$, we obtain the following expression for $\gamma^{c}$:


(9) $$\gamma^{c}=\;\frac{N_{u}-N_{g}\cdot n_{\min}^{*}}{N_{u}}.$$

**Fig. 4.**
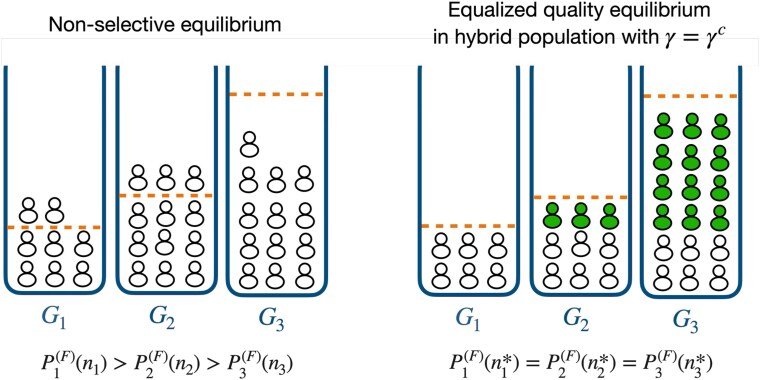
Distribution of a population over three common goods. The dashed horizontal lines within each good represent the number of users at each server at the equalized quality equilibrium, with $n_{1}^{*}<n_{2}^{*}<n_{3}^{*}$, for a given total population size $N_{u}$. On the left, the nonselective equilibrium given by Eq. 2 makes $G_{1}$ and $G_{2}$ overused and $G_{3}$ underused. On the right, selective individuals in a hybrid population are shown with solid filling. The system is chosen at $\gamma =\gamma^{c}$, i.e. the minimum proportion of selective individuals that allows the population to achieve equalized quality. Because of that, only nonselective individuals use $G_{1}$ in the hybrid system. At the equalized quality equilibrium, nonselective individuals use the three goods at the same rate. This is achieved through selective individuals avoiding $G_{1}$ and distributing over $G_{2}$ and $G_{3}$ respecting Eq. 10.

An illustration of what happens under $\gamma =\gamma_{c}$ is given in Fig. 4. If $\gamma <\gamma^{c}$, the good(s) associated with $n_{\min}^{*}$ will necessarily have a usage larger than that value, thus never achieving equalized quality. To obtain equalized quality under $\gamma =\gamma^{c}$, selective individuals will have to distribute themselves among the remaining common goods by choosing the following values of tolerance to failure:


(10) $$\frac{T_{i}^{\left(2\right)}}{\sum_{j}T_{j}^{\left(2\right)}}\;=\;\frac{n_{i}^{*}-n_{\min}^{*}}{N_{u}-N_{g}\cdot n_{\min}^{*}},$$

thus forcing the remaining $(1-\gamma^{c})N_{u}$ nonselective individuals to distribute equally between common goods.

## Adaptive tolerance to common good failure

Let us consider self-interested individuals with selective tolerance values who are averse to the usage of common goods with lower quality and higher probability of failure. These individuals may adapt their tolerance to common good failure to minimize reliance on such goods. A population of such individuals is hypothesized to attain the equalized quality distribution $n_{i}^{*}$ given that the interests of individuals are aligned, as explained in the previous sections. We introduce a learning method, formally defined in the “Materials and methods”. In this method, individuals collect information on their previous usage, estimate common good-specific failure probabilities and adapt their tolerance to failure under each common good accordingly. This method is evaluated within Internet service access in the following subsections using the parameters defined in the “Materials and methods”. We further analyze a population of individuals of one single type with adaptive tolerance values, concluding in the “Materials and methods” that the equilibrium point defined by values $n_{i}^{*}$ corresponding to Definition 1 and $T_{i}=T_{i}^{*}$ given by Theorem 1 are asymptotically stable.

### Evaluation of adaptive tolerance in Internet services

**Fig. 5.**
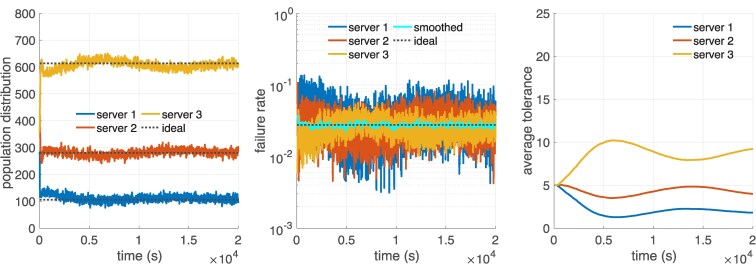
Simulation of a population of 1,000 users using a WSLS strategy with adaptive tolerance to common good failure accessing three servers with different capacity and delay values. We show the evolution of the population distribution, server-specific failure probability, and average tolerance under a system workload of ρ=0.75. Tolerance values are learned by each user independently. The value associated with “smoothed” curve reports the average of the low-pass-filtered system-level failure probability, taken over the last 10% of samples. See “Materials and methods” for details on the simulator, the adaptive tolerance method, and the used parameters.

We evaluate the performance of populations composed of individuals with adaptive tolerance to common good failure, whose results we present in Fig. 5. In the first panel, we observe that the population quickly reaches a distribution which has clear stochastic fluctuations and subtle long-term oscillations, but which is close to an equilibrium state. The oscillations are smooth and dampen over time while the distribution reaches the ideal distribution with equalized quality, as is shown by our analysis in Fig. 5. Furthermore, this is in line with the asymptotical stability of the population distribution equilibrium which was shown in the “Materials and methods”.

The second panel in Fig. 5 shows that the overall average system failure probability stabilizes quickly at 0.0265, within a 4% error margin of the theoretical ideal distribution for those conditions. Nonetheless, server-specific failure probabilities show some oscillations which are analyzed later. Servers 1 and 2 have lower capacity and tend to be overused in the early stages of the evolution. This is later corrected by the individual’s independent learning process, which eventually overshoots the tolerance at server 3, thus leading to small self-correcting oscillations—this can be seen in the third panel in Fig. 5. In the Supporting Information and figure S4, we consider the evolution of the population with larger values of the initialized tolerance to failure, showing that this extends the time required to reach equilibrium without affecting the final values of the equilibrium distribution state.

**Fig. 6.**
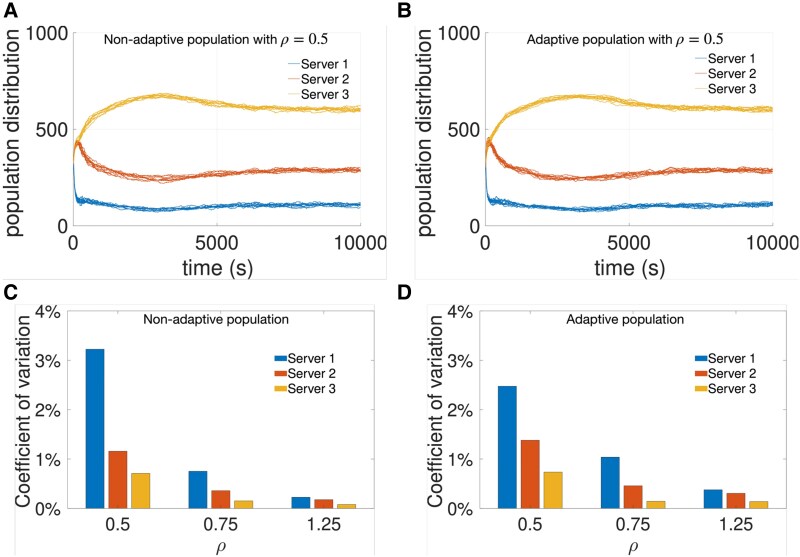
Evolution of the population distribution across 15 simulation runs of a population using the WSLS strategy with nonadaptive (left) and adaptive (right) tolerance to common good failure. A, B) show the evolution of the population distribution for ρ=0.5, while C, D) present the coefficient of variation over the final 2000s of each trajectory. Users are initially distributed at random over the three servers $G_{1}$, $G_{2}$, and $G_{3}$. Nonadaptive individuals have a set tolerance to 5 failures, whereas adaptive individuals start from that and learn the tolerance values. See “Materials and methods” for details on the simulator, the adaptive tolerance method, and the used parameters.

To understand the observed oscillations, Fig. 6 presents a set of simulations for the same population, each initialized with a random distribution over the servers. The first row shows that differences between the overlapping curves within each plot are minimal, despite the random initialization, indicating that the population distributions follow stable trajectories. This is further supported by the consistently low coefficients of variation across all trajectories, as shown in the second row. Nonetheless, higher system workload values produce smaller oscillations across all servers when compared to the results from lower workload values, such as those observed in the first row.

### Evaluation for changing workload

**Fig. 7.**
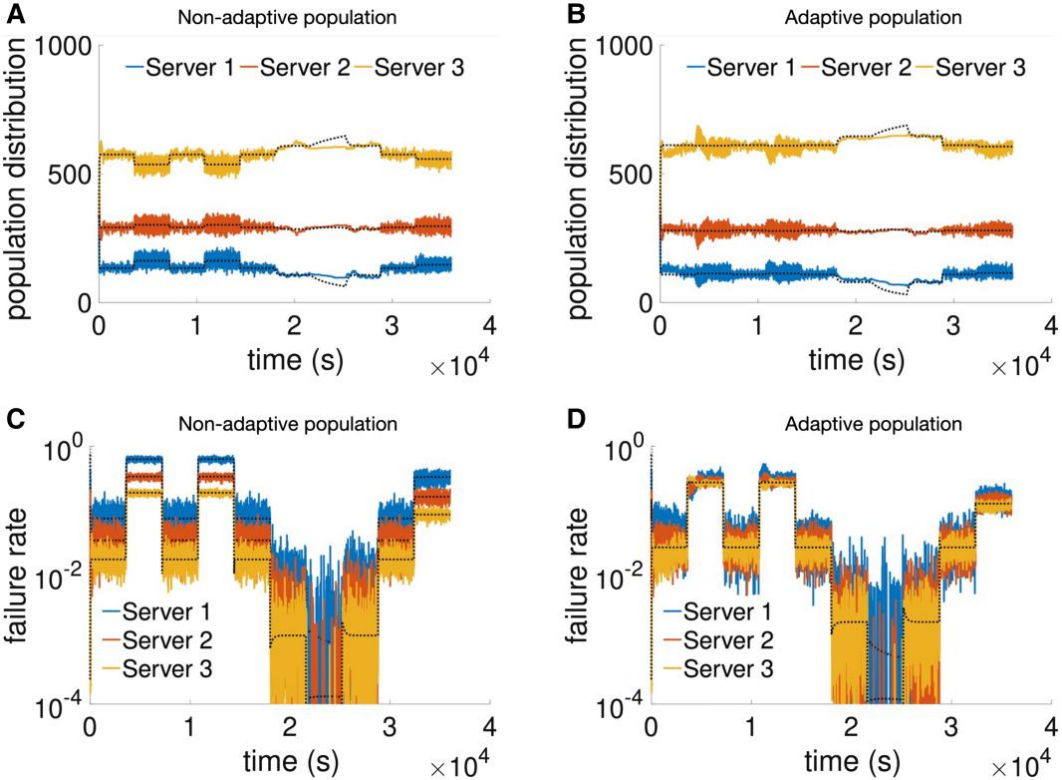
Simulation of a population using a WSLS strategy with nonadaptive (left) and adaptive (right) tolerance to common good failure under a system workload changing in time. A, B) show the evolution of the population distribution, while C, D) present the server-specific failure probability. The system workload (*ρ*) is set to switch every hour according to the following sequence: 0.75, 1.25, 0.75, 1.25, 0.75, 0.5, 0.25, 0.5, 0.75, and 1. Nonadaptive individuals have a fixed tolerance to 5 failures, whereas adaptive individuals learn their values starting from that. The ODE results are obtained applying Eq. 3. The ODEs of adaptive populations are obtained by setting tolerance values guaranteeing the idealized convergence towards equalized probability of failure. See “Materials and Methods” for details on the simulator, the adaptive tolerance method, and the used parameters.

We studied the evolution of the population distribution and server-specific failure probabilities when the workload of the system changes in time. For this, we have simulated the workload changing every hour to a value between ρ=0.25 and ρ=1.25, as reported in Fig. 7. The population distribution and the failure probabilities quickly change after workload values are switched. In the nonadaptive populations, server-specific failure probabilities stabilize at different values. These values can be well differentiated, even when overloading at ρ=1.25, where average failure probability is at least 20%.

In comparison, the adaptive population reaches remarkably identical failure probabilities between servers, with only small differences emerging from them. The small differences are likely to come from the fact that we have initialized tolerance values at 5, thus meaning that their learned values are limited between 1 and 13, with the sum of them being fixed at 15. This is a limit to the maximum difference between the learned tolerance values at each of the three servers, which are slightly visible for large workload values. Nonetheless, as noted before, the results obtained are remarkably close to equalized probability of failure between servers, and valid under a wide range of system workload values and quick dynamic changes.

The theoretical ODE results typically match the average behavior of the simulation results, apart from the intervals of extremely low system workload ρ=0.25. In such cases, the simulated population distribution changes much more slowly than the ODE predictions. This is likely associated with the fact that the ODEs assume immediate adaptation of failure rates to changes in the population distribution or in system workload values, whereas simulations take time to achieve those values. This is in line with what is observed in Fig. 3, where the population distribution in the simulation takes time to catch up with the ODE values for lower system workload values.

## Discussion

In this work, we propose an extension of the Win-Stay, Lose-Shift strategy for individuals using common goods with different options available to them. We show that its adoption leads to the self-organization of ideal distribution of usage with equalized quality, inspired by the ideal free distribution (IFD), effectively preventing the overuse or overconsumption of individual resources and a “tragedy of the commons.” Remarkably, this emerges without any need for information exchange between users, central coordination, or externally imposed control policies—features commonly relied upon in previous solutions. The WSLS strategy therefore offers a strikingly simple, low-information, robust, and fully decentralized approach. Its broad applicability to the governance of both natural and human-made common goods suggests important political and institutional implications, which we explore in this study.

In its simplest form, the WSLS strategy requires no memory of past usage—individuals act solely based on their most recent outcome. Even this minimal version leads to a more distributed usage of common goods when compared to scenarios where individuals spend equal time in each common good. However, it is the introduction of selective tolerance to common good failure that enables populations of self-interested individuals to self-organize into an ideal usage distribution, as demonstrated by realistic simulations of adaptive behavior. The introduction of such adaptive individuals into hybrid populations—alongside nonadaptive individuals—increases the overall experienced quality. Remarkably, if adaptive individuals surpass a critical prevalence, the system can reach the ideal distribution with equalized quality. This is reminiscent of what is observed in behavioral experiments and modeling approaches of prosocial behavior, where introducing hardwired agents into a hybrid population can promote cooperative behavior (23), and virtual agents may trigger large-scale prosocial behavior in populations of humans-agent populations (24). We will explore this further in a follow-up paper.

We considered these developments in light of the server selection problem faced by mobile users accessing Internet services. A set of server selection strategies are explored in Ref. (21), under which usage probabilities at each server either depend on the current state of the network or, more simply, on its fixed parameters. However, contrary to the WSLS strategy, both require individuals to acquire and process global network information. A more decentralized approach is proposed in Ref. (22), where individuals aim to minimize their own average failure probability. However, this strategy involves frequent server shifting which is often costly, and it may not scale well with larger number of servers and the population size. These and other previously proposed strategies, surveyed in Ref. (19), may achieve equalized failure probabilities, or optimize individual or system failure probabilities. However, the WSLS strategy with adaptive tolerance to failure offers a distinct advantage in being fully decentralized, scalable, robust to dynamic load changes, and minimizing costly shifting. This makes it a strong candidate for mobile network selection strategy, with potential to improve overall network accessibility, even in hybrid populations using diverse strategies. Future work will extend evaluations to realistic experimental testbeds (21, 22, 25) and potentially develop platform implementations.

In biological foraging and grazing systems, populations often self-organize to equalize the quality of available common goods. This occurs, for example, when bumblebees selectively frequent different land patches based on flower density and nectar availability (26, 27), or when cooperative spider colonies grow and disperse in sizes that reflect the local availability of insect prey (28). As proposed in Ref. (4), such distributions can arise through repeated movement between resource options, allowing individuals to assess and compare their quality. Our results point to an alternative strategy that may evolve, one that remains effective even when resource quality can only be inferred through prolonged use rather than instantaneous inspection.

In the context of public transport usage, studies of behavioral patterns show that users tend to follow the same commuting route on a regular basis (29), even when facing small disruptions (30). Disruptions coming from overcrowding can be locally regulated through collective rerouting strategies (31), but individuals experiencing successive disruptions with their typical route may also decide to shift to a different option on a daily basis. This makes public transport usage a particularly relevant domain for exploring how WSLS-inspired strategies may be shaping individual behavior, offering insights for the design of scalable, decentralized mechanisms to regulate usage patterns.

A similar perspective applies to water supply management in urban areas. These are complex interconnected systems, where controlling mechanisms guarantee the continuous access to water resources, available in different water storage units (32). Population dynamics approaches have been used to design solutions that ensure reliable water access in dense urban areas, including some based on the principle behind the ideal free distribution (33). Upon system failures resulting in intermittent water supply, households develop adaptive strategies (34), a pattern more generally observed in climate-related disasters (35). WSLS strategies provide a general decentralized and adaptive mechanism for distributing resource usage in such cases, highlighting their broader relevance for governing both natural and human-made resource systems.

## Materials and methods

### Proof of Theorem 1

Proof. The system of equations defined by Eq. 4 characterizes the equilibrium conditions of a heterogeneous population. This means that the presence of a type *k* at any good can be written as a function of $n_{1k}$:

(11) $$n_{\;jk}=n_{1k}\cdot \frac{P_{1}^{\left(F\right)}\left(n_{1}\right)}{T_{1}^{\left(k\right)}}\cdot \frac{T_{j}^{\left(k\right)}}{P_{\;j}^{\left(F\right)}\left(n_{\;j}\right)}.$$

Therefore, the total number of individuals $N_{u}^{\left(k\right)}$ of type *k* is equal to the following at equilibrium:

(12) $$N_{u}^{\left(k\right)}=\sum_{j}n_{\;jk}=n_{1k}\frac{P_{1}^{\left(F\right)}\left(n_{1}\right)}{T_{1}^{\left(k\right)}}\sum_{j}\frac{T_{j}^{\left(k\right)}}{P_{\;j}^{\left(F\right)}\left(n_{\;j}\right)},$$

which can be rearranged as:

(13) $$n_{1k}=N_{u}^{\left(k\right)}\cdot \frac{T_{1}^{\left(k\right)}/P_{1}^{\left(F\right)}\left(n_{1}\right)}{\sum_{j}T_{j}^{\left(k\right)}/P_{\;j}^{\left(F\right)}\left(n_{\;j}\right)}.$$

This relation is not valid just for i=1 but for any *i*. Therefore, we can represent $n_{ik}$ at equilibrium the following way:

(14) $$n_{ik}=N_{u}^{\left(k\right)}\cdot \frac{T_{i}^{\left(k\right)}/P_{i}^{\left(F\right)}\left(n_{i}\right)}{\sum_{j}T_{j}^{\left(k\right)}/P_{\;j}^{\left(F\right)}\left(n_{\;j}\right)}.$$

We now hypothesize that there is a set of tolerance vectors for which the population achieves the distribution with equalized quality $n_{i}^{*}$ (see Definition 1). In that case, the tolerance vector of each type will relate to their distribution in the following way:

(15) $$n_{ik}=N_{u}^{\left(k\right)}\cdot \frac{T_{i}^{\left(k\right)}}{\sum_{j}T_{j}^{\left(k\right)}}.$$

However, $n_{i}^{*}$ can be attained by different distributions of types over the goods. We thus sum over all types *k* to relate the population distribution and the tolerance vectors in the equalized quality state:

(16) $$n_{i}^{*}=\sum_{k=1}^{N_{t}}N_{u}^{\left(k\right)}\cdot \frac{T_{i}^{\left(k\right)}}{\sum_{j}T_{j}^{\left(k\right)}}.$$

Therefore, any combination of types with tolerance vectors $T_{i}^{\left(k\right)}$ and size $N_{u}^{\left(k\right)}$ that respects the equation above will lead to an equalized quality equilibrium $n_{i}^{*}$.

### Simulator

The model presented above was applied to the server selection problem in Internet services, and validated by comparing its results with a discrete-event simulator developed in Matlab. The simulator reproduces the arrival of requests from independent individuals to chosen servers, and tracks how individuals change server over time as a response to the observed performance of the server they use. In Table 1, we present the parameters considered in the model and the values we used in the simulator.

**Table 1.** Parameters used in the simulator of Internet access.

Notation	Parameter	Values
$N_{u}$	Number of users	1,000
$N_{g}$	Number of servers available	3
$G_{i}$	Servers	$\left\{G_{1},G_{2},G_{3}\right\}$
$\mu_{i}$	Service capacity of server $G_{i}$	{100,200,400} (servs/s)
*μ*	System service capacity	$\sum_{i}\mu_{i}=700$ (servs/s)
*ρ*	System workload	0.25;0.5;0.75;1;1.25
$\lambda_{u}$	User service request rate	$\rho \cdot \mu /N_{u}$ (reqs/s)
$c_{i}$	Number of processors of sever $G_{i}$	1
$k_{i}$	Buffer size of server $G_{i}$	10 reqs
$d_{i}$	Time between individuals and server $G_{i}$	{10,20,30} ms
*τ*	Service timeout	100 ms
$T_{i}^{\left(k\right)}$	Tolerance of type *k* on server $G_{i}$	1;5;adaptive
$T_{0}$	Initialized adaptive tolerance	5;10
$x_{0}$	Initialized estimated failure probability	0
*β*	Learning rate	0.10

For free parameters, we display the values used in the evaluations, whereas for dependent parameters we denote their dependence.

We consider a population of $N_{u}$ mobile users, often described as “user equipment” (UE) in the literature. Each user connects to the same base station (BS), which is attached to the backhaul (BH) through which a set of $N_{g}$ servers can be reached. As previously described, each individual issues on average $\lambda_{u}$ requests per second (reqs/s) to their server of choice and at each time there are $n_{i}$ individuals submitting requests on server $G_{i}$. The time delay between a request being sent and its arrival at the chosen server $G_{i}$ is denoted as $d_{i}$. These are considered to be the same for all individuals as they connect to the same base station. In the results presented here, the delays $d_{i}$ were considered to be deterministic. We explored the case where the delays are random variables following different distributions, and observed that these led to practically identical results for the dynamics and equilibria.

Each server $G_{i}$ is modeled as a Markovian queuing system of the general form $M/M/c_{i}/k_{i}$. In such systems, requests from each user arrive independently following a Poisson process with average rate given by $\lambda_{u}$, so that the aggregate process of arrivals at server $G_{i}$ is a Poisson process as well with an average server arrival rate of $n_{i}\lambda_{u}$. Requests are queued and processed in first come, first served order (FCFS), according to the availability of processors. The service time of arriving requests at server $G_{i}$ follows an exponential distribution with average value $\mu_{i}^{-1}$, where $\mu_{i}$ denotes the capacity of the server, i.e. the average number of requests they serve per second (servs/s). The number of available processors in the server is given by $c_{i}$, each of which can take one request at a time. We denote $k_{i}$ as the buffer size, with $k_{i}-c_{i}$ being the maximum number of requests waiting to be served.

The system workload is denoted as *ρ* and defines the ratio between the total population request rate $N_{u}\lambda_{u}$ and the total service capacity of the system $\mu =\sum_{i}\mu_{i}$. Since the capacity of servers and the population size is constant in the simulations, the system workload is varied by choosing the user service request rate as $\lambda_{u}=\rho \cdot \mu /N_{u}$. For example, to set the system workload as ρ=1, we set $\lambda_{u}=1\cdot 700/1,000=0.7$ reqs/s.

At the beginning of the simulation, individuals select one server each, uniformly at random. Each individual starts to send a Markovian process of requests to the chosen server as described above. The simulator tracks individual failures, i.e. requests which are lost because they arrive when the buffer size is full or those whose return delay (counted as the sum of the delay between individual and server and back and the service time at the server) exceeds the set timeout *τ*. The results shown in “Application to Internet services” were obtained by considering that, after experiencing a single failed request, an individual shifts to another server at random. However, in the evaluations shown in “Adaptive tolerance to common good failure,” each individual *h* has their own set of tolerance values, $T_{i}^{\left(h\right)},\;i=1,\ldots ,N_{g}$, one for each of the available servers. In those cases, the simulator counts individual failures. When the failure count of an individual *h* sending requests to server $G_{i}$ hits the tolerance value $T_{i}^{\left(h\right)}$, the individual shifts to another server, and the failure counter is reset. The next server to be used is selected uniformly at random. In the Supporting Information and Fig. S3, we show that choosing servers proportionally to the tolerance values leads to similar resulting equilibria. In the next section, we describe the adaptive tolerance method.

In this simulator, we track the evolution in time of both the number of individuals using each server, and the server-specific and average failure probabilities. In the case of adaptive tolerance to common good failure, the simulator additionally tracks the evolution in time of the average tolerance in the population.

### Adaptive tolerance method

We propose an adaptive tolerance method relying only on one’s previous experiences with usage of the common goods, thus avoiding considering communication or direct coordination between different individuals. Individuals perform an assessment of their own success rates and adapt their tolerance values accordingly.

For each focal individual *h* with adaptive tolerance:

- Define a vector for the estimated usage failure probability under each common good $x^{\left(h\right)}=(x_{1}^{\left(h\right)},\ldots ,x_{N_{g}}^{\left(h\right)})$. Initialize it with values $x_{0}$ for all common goods.
- Define a vector for the strategic tolerance to failure under each common good $T^{\left(h\right)}=(T_{1}^{\left(h\right)},\ldots ,T_{N_{g}}^{\left(h\right)})$. Initialize it with values $T_{0}$ for all common goods.
- The individual will choose a common good $G_{i}$ at random and attempt to use it repeatedly until $T_{i}^{\left(h\right)}$ failures are achieved. We denote *R* as the number of usage attempts until the $T_{i}^{\left(h\right)}$ failures are achieved.
- The individual will update the estimated usage failure probability under that common good $x_{i}^{\left(h\right)}$ considering both the previous estimation and the experienced rate $T_{i}^{\left(h\right)}/R$:
  $$x_{i}^{\left(h\right)}⟵(1-\beta )\cdot x_{i}^{\left(h\right)}+\beta \cdot T_{i}^{\left(h\right)}/R,$$
  where *β* is the learning rate.
- The individual will update the vector of strategic tolerance $T^{\left(h\right)}$ based on the information on vector $x^{\left(h\right)}$. Considering $l=\arg\;\min_{j}x_{j}^{\left(h\right)}$, they update if $x_{l}^{\left(h\right)}<x_{i}^{\left(h\right)}$ and $T_{i}^{\left(h\right)}>1$:
  $$\begin{array}{rl} & T_{i}^{\left(h\right)}⟵T_{i}^{\left(h\right)}-1\\ & T_{l}^{\left(h\right)}⟵T_{l}^{\left(h\right)}+1. \end{array}$$
- The individual will shift to one of the other common goods randomly and restart the usage phase.

### Equilibrium and stability

The state of a system with a single type of individual is described by the population vector $\vec{n}$ and the tolerance vector $\vec{T}$:


$$\vec{s}=[\vec{n},\vec{T}].$$

The ODEs defining the dynamics of the system are of the form


$$\frac{ds_{i}}{dt}\;=g_{i}(t),$$

with $g_{i}(t)$ being a nonlinear function of the whole state vector at time *t*, i.e. the tolerance values and population distribution over the common goods. We define $g_{i}=\nu_{i}$ for the ODEs corresponding to $\vec{n}$, and $g_{N_{g}+i}=\xi_{i}$ for the ODEs corresponding to $\vec{T}$. In particular, the ODEs for $\vec{n}$ can be simply obtained from Eq. 3 with one single type of individual, and the ODEs for $\vec{T}$ depend on the specific adaptive tolerance method considered.

Notice that, for $\vec{n}$, each ODE has an attractor at the equilibrium point because the ODE can be expressed as


$$\nu_{i}=\;\frac{dn_{i}}{dt}\;=-\lambda_{u}n_{i}\frac{P_{i}^{\left(F\right)}(n_{i})}{T_{i}}\;+\;\frac{1}{N_{g}-1}\sum_{\;j\neq i}\lambda_{u}n_{j}\frac{P_{j}^{\left(F\right)}(n_{j})}{T_{j}}.$$

Given all variables are positive, the first term is negative while the second is positive (they represent outgoing and incoming population flows, respectively). When deviating a little from the equilibrium $n_{i}^{*}$, both terms must decrease with $n_{i}$, due to the fact that an increase in $n_{i}$ is balanced by decreases in the other populations. Hence, there must exist an interval around the equilibrium point in which $\nu_{i}$ is negative if $n_{i}$ is higher than the equilibrium and positive if $n_{i}$ is lower than it (remember that, at the equilibrium, $\nu_{i}=0$).

ODEs for $T_{i}$ are of the following kind:


$$\xi_{i}=\frac{dT_{i}}{dt}=h(\vec{n}),$$

where we considered the simplifying assumption that changes in $T_{i}$ are provoked only by imbalances on the failure probabilities at different common goods, which in turn are functions of the population distribution and not of the tolerance values. Small variations of $T_{i}$ in an interval around the equilibrium point will perturb $n_{i}$ positively since higher $T_{i}$ leads to higher $n_{i}$. This, in turn, will cause a negative feedback on $T_{i}$, since the higher $n_{i}$ goes, the higher the failure probability at that common good, and the more $T_{i}$ will decrease. Therefore, $\xi_{i}<0$ if $T_{i}>T_{i}^{*}$ and, $\xi_{i}>0$ if $T_{i}<T_{i}^{*}$.

Let us assume that an equilibrium point exists for $\vec{s}$, and denote it by $\vec{s^{*}}$. Next we prove the asymptotic stability of state trajectories by using the transformed state $\vec{z}$ = $\vec{s}-\vec{s^{*}}$, whose stability point is $\vec{0}$. To do so, we need to identify a Lyapunov scalar function *V* of the state components, with the properties of being positive definite in an interval around the equilibrium point and with a negative definite derivative with respect to time.

Let us consider the following (globally) positive definite function:


(17) $$V\left(\vec{z}\right)\;=\sum_{i=1}^{2N_{g}}z_{i}^{2}=\sum_{i=1}^{N_{g}}{\left(n_{i}-n_{i}^{*}\right)}^{2}\;+\sum_{i=1}^{N_{g}}{\left(T_{i}-T_{i}^{*}\right)}^{2}.$$

The derivative of *V* is expressed as follows:


(18) $$\frac{dV\left(\vec{z}\right)}{dt}\;=\sum_{i=1}^{2N_{g}}\frac{\partial V}{\partial z_{i}}\frac{dz_{i}}{dt}\;=2\left(\sum_{i=1}^{N_{g}}z_{i}\nu_{i}+\sum_{i=1}^{N_{g}}z_{N_{g}+i}\xi_{i}\right).$$

From what derived above, there is an interval around $\vec{0}$ for which when $z_{i},\;i\in \{1,\ldots ,2N_{g}\}$ is positive, $\nu_{i}$ will be negative, and vice versa. Hence, product $z_{i}\nu_{i}$ is zero at the equilibrium and negative in the region around the equilibrium. Moreover, there is an interval around the equilibrium point in which $\xi_{i}$ is either 0 or has the opposite sign of the deviation of $T_{i}$. Therefore, there must exist a $2N_{g}-$dimensional region around the equilibrium $\left[\vec{n^{*}},\vec{T^{*}}\right]$ in which all terms in the first sum of the expression for $\frac{dV}{dt}$ are negative, and the terms in the second part are nonpositive. Of course, the derivative is exactly zero at the equilibrium point, because so are all ODEs $\nu_{i}$ and $\xi_{i}$. Hence $\frac{dV}{dt}$ is negative definite and the equilibrium point is asymptotically stable.

## Supplementary Material

pgaf374_Supplementary_Data

## Data Availability

All supporting code is available on GitHub (36): https://github.com/paolocastagno/SelfOrganizationOfCommonGoodsUsage.
