# Supplementary material for: Self-organization of common good usage and an application to Internet services
Source: PNAS Nexus. 2025 Dec 1;4(12):pgaf374. doi: 10.1093/pnasnexus/pgaf374 (PMC12707203; doi:10.1093/pnasnexus/pgaf374)
Supplement: pgaf374_Supplementary_Data [file pgaf374_supplementary_data.pdf]

Supporting Information for

Self-organisation of common good usage and an application to Internet services

Diogo L. Pires, Vincenzo Mancuso, Paolo Castagno, and Marco Ajmone Marsan

Corresponding Author name.
E-mail: diogo.l.pires@gmail.com

This PDF file includes:

Supporting text
Figs. S1 to S4

Supporting Information Text

In the supplementary material we explore alternative assumptions on the model and simulations used. We explore a variation of fixed values of tolerance to failure and system workloads, as well as different assumptions about probability of shifting commons. These results are sustained by extra simulations.

The effect of higher tolerance to failure

In this section, we explore the effect of considering higher values of tolerance to failure. We consider the same setting explored in “Application to Internet services" of the main text, with the exception that here we choose a different system workload of $\rho = 0.75$, and explore different values of tolerance $T = \{1, 2, 20\}$ for comparison. Note that under $T = 1$, the original WSLS strategy explored in that section is recovered. The results are presented in figure S1.

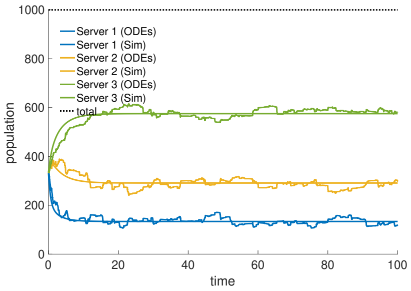

(a) Population distribution evolution under $T = 1$.

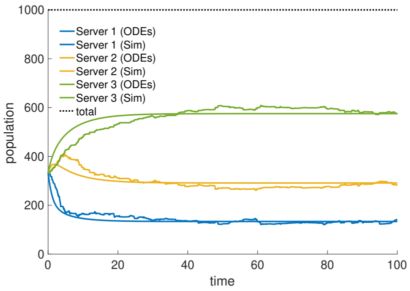

(b) Population distribution evolution under $T = 2$.

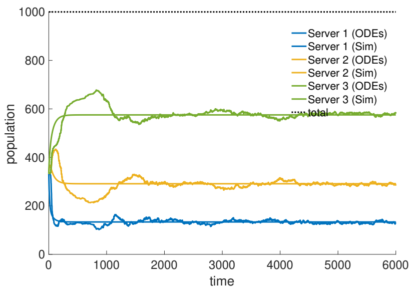

(c) Population distribution evolution under $T = 20$.

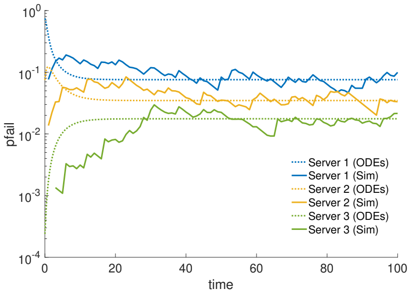

(d) Evolution of failure probability under $T = 1$.

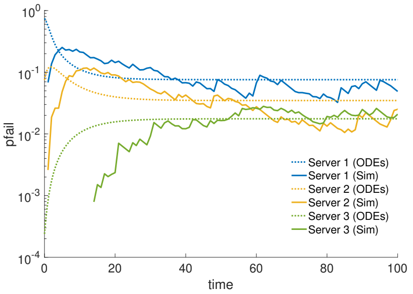

(e) Evolution of failure probability under $T = 2$.

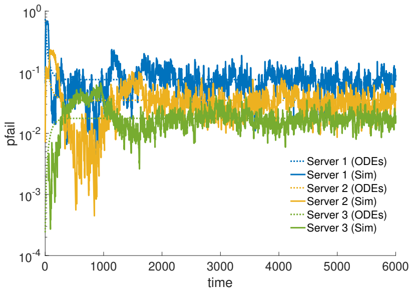

(f) Evolution of failure probability under $T = 20$.

Fig. S1. Simulation of a population of 1000 users using a WSLS strategy with different tolerance to failure, accessing three servers with different capacity and latency values with system workload of $\rho = 0.75$. We show the evolution of the population distribution and server-specific failure probability. The results presented here are parallel to those of figure 3 of the main text, with the difference that they are for a fixed system workload and for different tolerance values. See main text, particularly “Materials and Methods" for details on the simulator and table 1 for the used parameters.

We start by noting that under $T = 1$ (see figures S1(a) and S1(d)), there are substantially more and quicker stochastic fluctuations of the population distribution and less fluctuations of the server-specific failure probabilities than those observed under lower workload (see figures 3(a) and 4(d) of the main text). This is consistent with what was already noted on the main text.

Moreover, we observe that increasing the tolerance values has the effect of extending the timescale at which the system evolves. We observe in figures S1(b) and S1(e) that the fluctuations are less frequent. In figures S1(c) and S1(f), a different time interval had to be considered so that we would understand the long term behaviour of the curves. As it can be seen in those figures, the long-term behaviour of the server-specific failure probabilities shows

The effect of higher tolerance to failure under different system workload

In this section, we consider the fixed value of tolerance to failure $T = 5$ and explore the system workloads of $\rho = 0.5, 0.75, 1.25$, as used in figure 3 of the main text. We present these results in figure S2. Comparing the results under $\rho = 0.5$ with $T = 5$ (see figures S2(a) and S2(d)) with those for the same workload when $T = 1$ (see figures 3(a) and 3(d) of the main text), we see once again that higher tolerance values extend the timescale at which the population distribution changes. This makes both the distribution and server-specific failure probabilities taking longer to stabilise.

Under larger system workload values of $\rho = 1, 1.25$, the system achieves its equilibrium distribution and server-specific failure probabilities quickly and with stochastic fluctuations of the same order as under $T = 1$. In this context, because failures are much more frequent, the system is able to self-organise quickly and reach the equilibrium distribution under higher tolerance values.

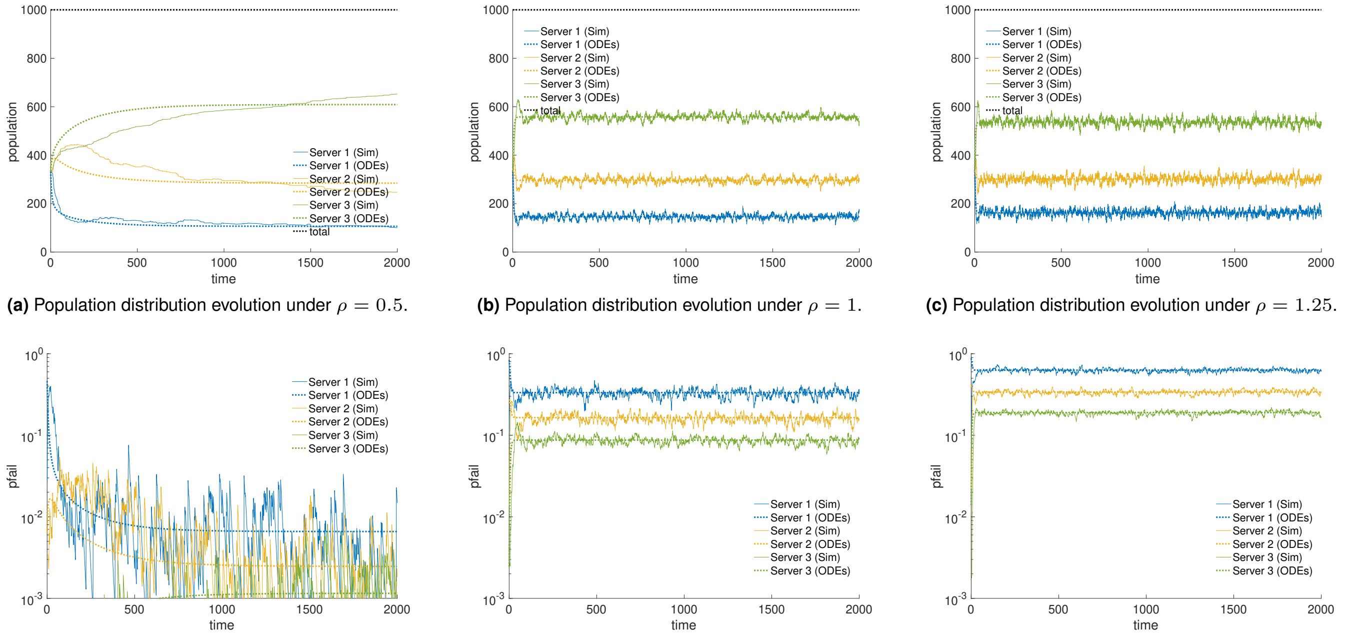

(a) Population distribution evolution under $\rho = 0.5$.
(b) Population distribution evolution under $\rho = 1$.
(c) Population distribution evolution under $\rho = 1.25$.
(d) Evolution of failure probability under $\rho = 0.5$.
(e) Evolution of failure probability under $\rho = 1$.
(f) Evolution of failure probability under $\rho = 1.25$.

Fig. S2. Simulation of a population of 1000 users using a WSLS strategy with tolerance to 5 failures ($T = 5$) accessing three servers with different capacity and latency values. We show the evolution of the population distribution and server-specific failure probability for different system workload values. The results presented here are parallel to those of figure 3 of the main text, with the difference that they are obtained for a tolerance value of $T = 5$, instead of $T = 1$. See main text, particularly "Materials and Methods" for details on the simulator and table 1 for the used parameters.

Shifting proportional to tolerance

Leaving behind the assumption that shifting to a new common good happens uniformly may have an impact. In the context of a population where individuals hold different values of tolerance to failure to each common good $T_i^{(k)}$, we may consider tolerance as a possible measure of the interest in that common good. As such, let us consider the dynamics obtained when upon shifting, individuals choose the new common good proportionally to the tolerance value. This leads to the following dynamical equations:

$$\dot{n_{ik}} = -\lambda_u \cdot n_{ik} \cdot \frac{P_i^{(F)}(n_i)}{T_i^{(k)}} + \sum_{j \neq i} \lambda_u n_{jk} \cdot \frac{P_j^{(F)}(n_j)}{T_j^{(k)}} \cdot \frac{T_i^{(k)}}{\sum_{l \neq j} T_l^{(k)}}, \quad [1]$$

where equilibrium is reached at the solution to the following set of equations:

$$\frac{n_{1k} \cdot P_1^{(F)}(n_{1k})}{{T_1^{(k)}}^2 \left(\sum_{l \neq 1} T_l^{(k)}\right)} = \frac{n_{2k} \cdot P_2^{(F)}(n_{2k})}{{T_2^{(k)}}^2 \left(\sum_{l \neq 2} T_l^{(k)}\right)} = \ldots = \frac{n_{N_g k} \cdot P_{N_g}^{(F)}\left(n_{N_g k}\right)}{{T_{N_g}^{(k)}}^2 \left(\sum_{l \neq N_g} T_l^{(k)}\right)}. \quad [2]$$

If we consider shifting to the same common good to be possible, the equations simplify to the following:

$$\dot{n_{ik}} = -\lambda_u \cdot n_{ik} \cdot \frac{P_i^{(F)}(n_i)}{T_i^{(k)}} + \sum_{j \neq i} \lambda_u n_{jk} \cdot \frac{P_j^{(F)}(n_j)}{T_j^{(k)}} \cdot \frac{T_i^{(k)}}{\sum_{l} T_l^{(k)}} \quad [3]$$

leading to the following equilibrium equations:

$$\frac{n_{1k} \cdot P_1^{(F)}(n_{1k})}{{T_1^{(k)}}^2} = \frac{n_{2k} \cdot P_2^{(F)}(n_{2k})}{{T_2^{(k)}}^2} = \ldots = \frac{n_{N_g k} \cdot P_{N_g}^{(F)}\left(n_{N_g k}\right)}{{T_{N_g}^{(k)}}^2}. \quad [4]$$

This result means that that shifting proportional to tolerance leads to a simple alteration of the equilibrium equations and therefore to a different balance between population distribution, failure probability at each common good, and tolerance values of the the population. Therefore, the resulting population distribution for the same values of tolerance will be different under this variation of the WSLS strategy.

However, under the adaptive tolerance method proposed in this work, the final population equilibrium is expected to be the same. Adaptive tolerance values will be adjusted until equalised failure probabilities are reached in both versions of the strategy. The final equilibria will happen at different values of tolerance to failure respecting equation 4 with equalised failure probabilities.

Evolution under adaptive tolerance to failure under shifting proportional to tolerance

In this section we compare the evolution plots obtained under the adaptive tolerance method with uniform shifting with the evolution obtained under the same method with shifting proportional to the learned tolerance. We observe that the difference between the two is minimal, with the proportional shifting leading to oscillations with slightly higher amplitude. This may be due to fact that the initial conditions are further away from equilibrium in the second case than in the first. This leads to slightly larger smoothed average failure probability. The equilibrium values of population distribution, failure probability and average tolerance are similar but should be slightly different based on the theoretical conclusions of the previous section.

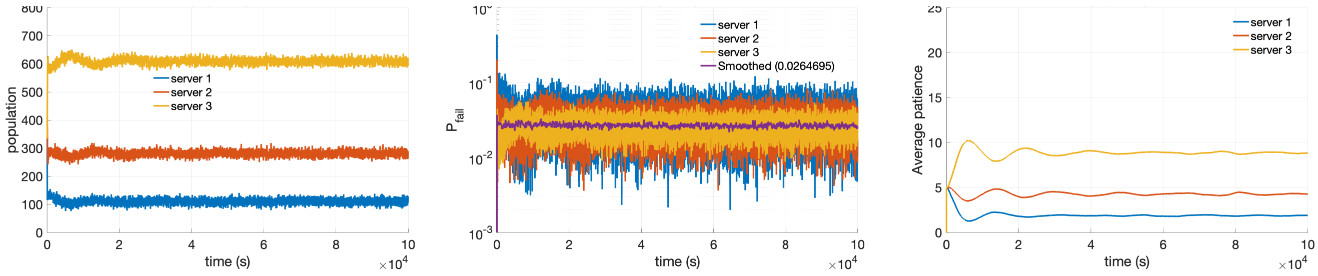

(a) Adaptive population following uniform shifting probability over the available serves.

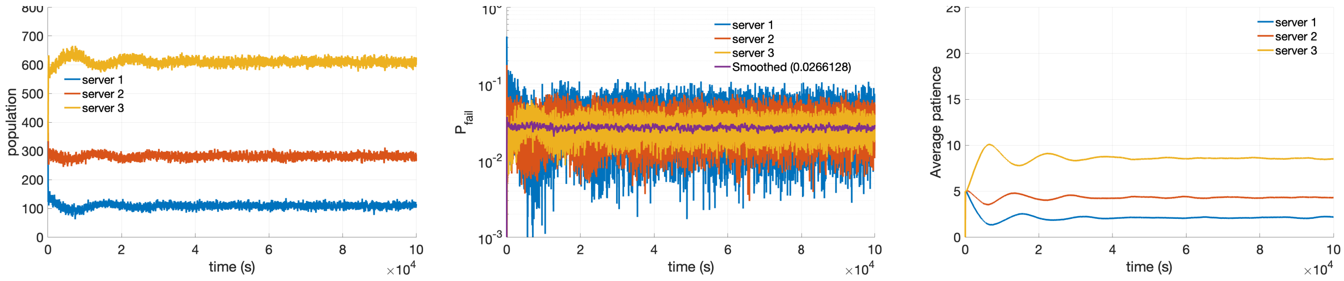

(b) Adaptive population following shifting probability over the available serves proportional to tolerance values.

Fig. S3. Simulation of a population of 1000 users using a WSLS strategy with adaptive tolerance to common goods failure accessing three servers with different capacity and delay values. We show the evolution of the population distribution, server-specific failure probability, and average tolerance under a system workload of $\rho = 0.75$. The results presented on figure S3(a) are the same as to those of figure 5 of the main text. This is repeated here for comparison with figure S3(b), where we consider individuals shift to a new common good proportionally to their tolerance. See main text, particularly “Materials and Methods" for details on the simulator and table 1 for the used parameters.

Adaptive tolerance method with higher total tolerance. We further consider different initialising values of tolerance and its effects on the evolution of the system. In this case, we have chosen $T_0 = 10$, instead of the $T_0 = 5$ used in remaining simulations of adaptive individuals. In this case, we observe that the extension of the long-term oscillations of all quantities shown in the figures are much more extended in time. Furthermore, their amplitude observed in server-specific failure probabilities and average tolerance are much larger. This should explain the slightly larger average failure probability observed in the overall system. Nonetheless, the values seem to tend in the direction of the equalised quality equilibrium, as all other simulations of entirely adaptive individuals, showing again the robustness of the adaptive method considered.

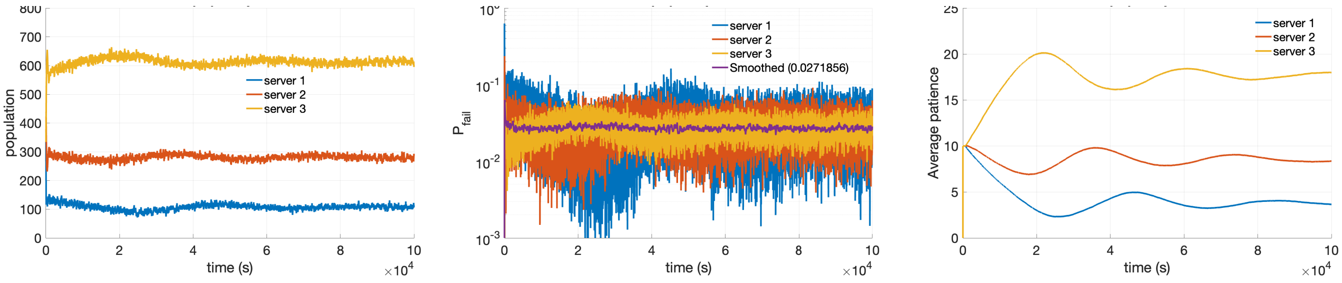

Fig. S4. Simulation of a population of 1000 users using a WSLS strategy with adaptive tolerance to common goods failure accessing three servers with different capacity and delay values. We show the evolution of the population distribution, server-specific failure probability, and average tolerance under a system workload of $\rho = 0.75$. The results presented here are parallel to those of figure 5 of the main text and figure S3, with the difference that we consider individuals to shift to a new common good proportionally to their tolerance, and that tolerance values are initialised at $T_0 = 10$, instead of $T_0 = 5$. See main text, particularly “Materials and Methods" for details on the simulator and table 1 for the used parameters.
